# Supplementary figures and images for: Genetic variants of Toll-like receptor 9 are associated with susceptibility to systemic lupus erythematosus in Han Chinese female patients
Source: PeerJ. 2025 Aug 13;13:e19847. doi: 10.7717/peerj.19847 (PMC12357549; doi:10.7717/peerj.19847)

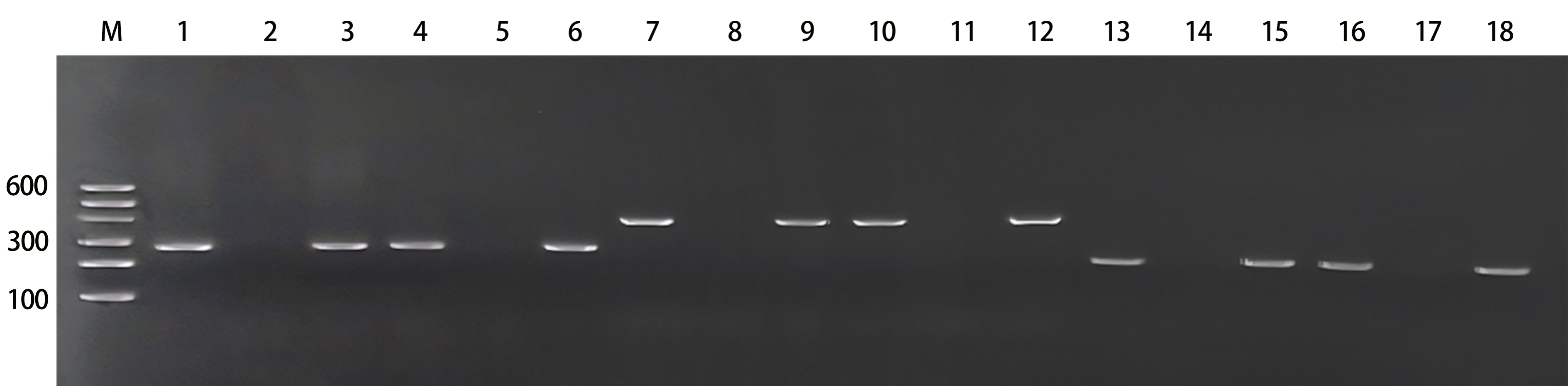

Supplement: Supplemental Information 1 — The band pattern for rs352139, Lanes 1 and 2 indicated GG genotype; Lanes 3 and 4 indicated AG genotype; Lanes 5 and 6 indicated AA genotype; The band pattern for rs352140, Lanes 7 and 8 indicated CC genotype; Lanes 9 and 10 indicated CT genotype; Lanes 11 and 12 indicated TT genotype. The band pattern for rs5743836, Lanes 13 and 14 indicated homozygous CC genotype; Lanes 15 and 16 indicated heterozygous TC genotype; Lanes 17 and 18 indicated homozygous TT genotype. Lane M shows a 600 bp DNA Marker. [file peerj-13-19847-s001.pdf]

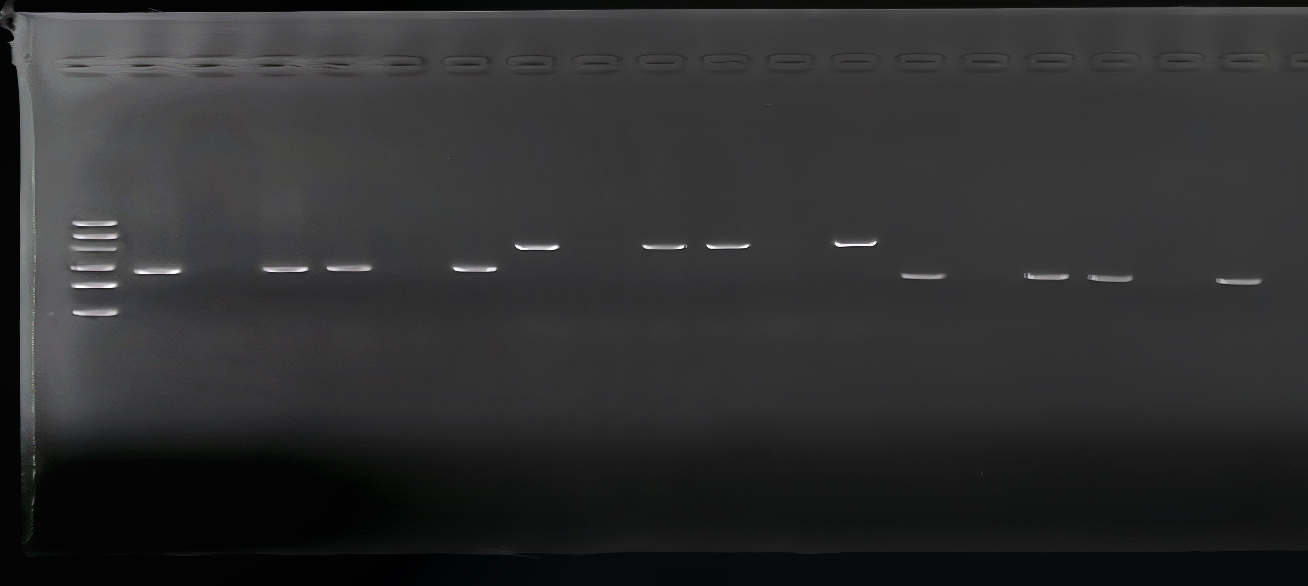

Supplement: Supplemental Information 2 [file peerj-13-19847-s002.jpg]
